# Emodin inhibiting neutrophil elastase‐induced epithelial‐mesenchymal transition through Notch1 signalling in alveolar epithelial cells

**DOI:** 10.1111/jcmm.15827

**Published:** 2020-09-15

**Authors:** Linshui Zhou, Rundi Gao, Huihua Hong, Xiaojuan Li, Jia Yang, Wei Shen, Zhen Wang, Junchao Yang

**Affiliations:** ^1^ Department of Respiration The First Affiliated Hospital of Zhejiang Chinese Medical University Hangzhou China; ^2^ Department of Pulmonary Function The First Affiliated Hospital of Zhejiang Chinese Medical University Hangzhou China; ^3^ The First Clinical Medical College Zhejiang Chinese Medical University Hangzhou China; ^4^ Department of Traditional Chinese medicine preparation The First Affiliated Hospital of Zhejiang Chinese Medical University Hangzhou China

**Keywords:** alveolar epithelial cells, Emodin, epithelial‐mesenchymal transition (EMT), neutrophil elastase (NE), Notch signalling, pulmonary fibrosis

## Abstract

The transition of alveolar type II epithelial cells into fibroblasts has been reported to cause and/or aggravate pulmonary fibrosis (PF), which is characterized by fibroblast proliferation, an enhanced production and accumulation of ECM (extracellular matrix), alveolar wall damage and functional capillary unit loss. Traditional Chinese medicine Emodin has been reported to inhibit TGF‐β‐induced epithelial‐mesenchymal transition (EMT) in alveolar epithelial cells through Notch signalling. In the present study, neutrophil elastase (NE, also known as ELA2) treatment promoted EMT, Notch1 cleavage (NICD/Notch1 ratio increase) and NICD nuclear translocation in RLE‐6TN cells and A549 cells. The promotive roles of NE treatment in these events were significantly reversed by Notch1 knockdown. Traditional Chinese medicine Emodin treatment remarkably inhibited the enzyme activity of NE, suppressed EMT, Notch1 cleavage and NICD nuclear translocation within RLE‐6TN and A549 cells, while NE treatment significantly reversed the effects of Emodin. Moreover, in RLE‐6TN, the effects of NE on EMT, Notch1 cleavage and NICD nuclear translocation were remarkably attenuated by Emodin treatment and more attenuated by the combination of Emodin and neutrophil elastase inhibitor Sivelestat or notch signal pathway inhibitor DAPT. In conclusion, we revealed the involvement of NE‐induced Notch1 cleavage in the functions of Emodin suppressing NE‐caused EMT in RLE‐6TN cells and A549 cells. This novel mechanism of Emodin inhibiting EMT might extend the application of Emodin in PF treatment.

**Abbreviations**
BAL: bronchoalveolar lavage
BLM: bleomycin
ECL: enhanced chemiluminescence
ECM: extracellular matrix
EMT: epithelial‐mesenchymal transition
IF staining: immunofluorescence staining
IPF: idiopathic pulmonary fibrosis
NE: neutrophil elastase
PF: pulmonary fibrosis
PPIs: Protein‐protein interactions
RLE‐6TN: rat lung epithelial‐T‐antigen negative

## 1 INTRODUCTION

Pulmonary fibrosis (PF), a pulmonary disease that occurs when lung tissue becomes damaged and scarred, is characterized by fibroblast proliferation, an enhanced production and accumulation of ECM (extracellular matrix), alveolar wall damage and functional capillary unit loss, finally resulting in respiratory failure.^1^, ^2^ Regarding the mechanism, the transition of alveolar type II cells into fibroblasts has been reported to cause and/or aggravate PF.^3^, ^4^ The present study exhibited the inhibitory effects of Emodin (the chemical structure was shown in Figure S1A), an anthraquinone mainly found in traditional Chinese medicines such as rhubarb, Polygonum multiflorum and Polygonum cuspidatum,^5^, ^6^ on PF through suppressing transforming growth factor β (TGF‐β)‐induced epithelial‐mesenchymal transition (EMT) within alveolar epithelial cells via Notch signalling.^7^ Further investigation on the potential mechanism of Emodin could possibly provide new strategies to treat pulmonary fibrosis.

The major biological role of Notch signalling pathway is to control the developmental fates of several cells.^8^, ^9^ Initially, Notch signalling pathway has been described as an essential pathway that suppresses cell differentiation via eliciting transcriptional repressors, including HES (hairy and enhancer of split) and HERP (HES‐related repressor protein) family genes.^10^ Moreover, various studies have demonstrated the important role of Notch signalling pathway within lung and airway fibrosis.^11^, ^12^ Recently, new evidence suggests that Notch signalling exerts a promotive effect on EMT in the process of development and tumorigenesis.^13^, ^14^ Hu et al ^15^ reported that selective Notch1 deficiency within mesenchymal cells led to fibrosis impairment because of deficient myofibroblast differentiation. In our previous study, we revealed that Emodin could modulate the gene expression profiles of Notch pathway‐associated factors and down‐regulate the Notch1 nucleus translocation. Silencing Notch1 can intensify the functions of Emodin suppressing TGF‐β1–caused EMT in RLE‐6TN cells.^16^ Considering these previous findings, investigating the factors that might mediate Emodin‐induced suppression on Notch1 nuclear translocation and the underlying mechanism is needed.

It is gradually accepted that cells might be involved in different biological functional modules,^17^, ^18^, ^19^ where different series of proteins interact with each other to form different building blocks, participating in different biological processes. Protein‐protein interactions (PPIs), which are widely used in the construction of biological networks, participate in protein transport and degradation, cell cycle progress, polarity, gene expression, DNA repair and many other biological processes.^20^ During the past decades, the availability of complete genome sequences has led to a paradigm shift from the study of individual proteins in an organism to large‐scale proteome‐wide studies of proteins, thereby constructing a large‐scale map of human protein interactions. PPI mapping can not only characterize a predicted function to unidentified proteins, but also provide information, such as interaction scopes, to guide further experiments. In the present study, the targets and PPIs of Emodin were obtained from ChEMBL (www.ebi.ac.uk/chembl/) and BindingDB (www.bindingdb.org) database retrieval and eight candidate proteins were selected (Figure S1B). BindingDB data are derived from PDB literature reports, patent information, PubChem BioAssay data and ChEMBL records, while ChEMBL data are from a variety of published literature. Among the candidate proteins, neutrophil elastase (NE, also known as ELA2) attracted our attention because of the vital role of neutrophils in PF.^21^, ^22^, ^23^, ^24^, ^25^ Since NE plays a promotive role in PF, we speculate that Emodin might interact with NE to affect Notch1 cleavage and Notch1 nuclear translocation, finally inhibiting EMT in RLE‐6TN and A549 cells.

Herein, we first examined the effects of different doses of NE on EMT markers and Notch1 protein levels. Next, Notch1 knockdown was generated in RLE‐6TN and A549 cells with or without NE treatment to investigate whether NE induces EMT through enhancing Notch1 cleavage and Notch1 nuclear translocation. Finally, the study co‐treated alveolar epithelial cells with Emodin and NE and then examined the EMT status and Notch1 cleavage. In summary, we demonstrate a novel mechanism of Emodin inhibiting EMT in alveolar epithelial cells via suppressing NE‐mediated Notch1 cleavage.

## 2 MATERIALS AND METHODS

### 2.1 Cell line and cell treatment

Rats alveolar type II epithelial cell line RLE‐6TN (ATCC^®^ CRL‐2300™) and human alveolar epithelial cell line A549 (ATCC^®^ CCL‐185™) were obtained from ATCC (Manassas, VA, USA). RLE‐6TN cells were cultured in Ham's F12 medium with 2 mM L‐glutamine supplemented with 0.01 mg/ml bovine pituitary extract, 0.005 mg/ml insulin, 2.5 ng/ml insulin‐like growth factor, 0.00125 mg/ml transferrin, 2.5 ng/ml EGF (90%) and 10% FBS. A549 were cultured in F‐12K Medium with 10% FBS. Cells were cultured at 37℃ in 5% CO_2_. The lung epithelial ell lines were authenticated using immunofluorescence staining (IF staining) with anti‐proSPC (ab90716; Abcam, Cambridge, UK).

Recombinant mouse NE protein (4517‐SE‐010) was obtained from R&D Systems (Minneapolis, MN, USA) and used to treat RLE‐6TN cells and A549 cells at concentrations of 50 nM, 100 nM or 200 nM. For Emodin treatment, Emodin (Sigma‐Aldrich, St. Louis, MI, USA) was firstly dissolved in DMSO (Sigma‐Aldrich) and further diluted to 10 µg/ml.^16^ The final DMSO concentration was less than 0.1% (v/v). Sivelestat (S7198; Sigma‐Aldrich), a neutrophil elastase inhibitor, was used to treat RLE‐6TN cells at a density of 100 µg/ml. The γ‐Secretase Inhibitors DAPT (ab120633, Abcam) was used as notch pathway inhibitor to RLE‐6TN cells at a density of 50 nM.

### 2.2 Immunoblotting analysis

Cells were transfected and/or treated and then lysed in lysis buffer (Beyotime, Shanghai, China). For nucleus protein isolation, cells were lysed by Nuclear and Cytoplasmic Protein Extraction Kit (Beyotime, China) according to the manufacture's instruction. The cell protein extracts (30 ~ 50 µg) were separated by sodium dodecylsulphate‐polyacrylamide gel electrophoresis (SDS‐PAGE) electrophoretically transferred onto polyvinylidene difluoride membranes, which were then blocked with 5% non‐fat dried milk in TBS‐T and then incubated with proper primary antibodies listed below: anti‐α‐SMA (14395‐1‐AP; ProteinTech, Rosemont, IL, USA), anti‐vimentin (60330‐1‐lg; ProteinTech), anti‐E‐cadherin (20874‐1‐AP; ProteinTech), anti‐Notch1 (ab83232; Abcam), anti‐C‐MYC (ab32072; Abcam), anti‐HES1 (ab71559; Abcam), anti‐HEY1 (ab22614; Abcam), anti‐GAPDH (60004‐1‐lg, ProteinTech, USA), anti‐Histone H3(17168‐1‐AP, ProteinTech) and anti‐β‐actin (60008‐1‐lg, ProteinTech). After washing with TBST buffer, the membranes were incubated with the appropriate secondary antibodies. Immunoreactive proteins were visualized using enhanced chemiluminescence (ECL) techniques.

### 2.3 Polymerase Chain Reaction (PCR)‐based analysis

Total RNA was isolated from target cells (transfected and/or treated) using the RNeasy mini kit (Qiagen, Chatsworth, CA) following the protocols. A GeneAmp RNA PCR Core Kit (Applied Biosystems, Tokyo, Japan) was used to convert the total RNA to cDNA following the protocols. The expression of Notch1 mRNA was then determined using SYBR Premix Ex Taq (TaKaRa, Shiga, Japan). The relative gene expression of Notch1 mRNA was calculated using the 2^−∆∆Ct^ method using the GAPDH expression as an initial control.

### 2.4 IF staining

For the authentication of RLE‐6TN and A549 cells and the detection of Notch‐1 nuclear translocation, IF staining was carried out using anti‐proSPC (ab90716, Abcam) and anti‐Notch1 (ab52627; Abcam), respectively, following the methods described in our previous study.^16^ DAPI (Beyotime, China) was used to stain nuclei before capturing images. The images were acquired using a fluorescence microscope (Nikon, Japan). The green fluorescence indicated proSPC or Notch‐1 protein, respectively, and the blue fluorescence indicated nuclei.

### 2.5 Determination of neutrophil elastase activity

The neutrophil elastase activity was examined using an Neutrophil Elastase Activity Assay Kit (Fluorometric, ab204730, Abcam) according to the methods described before.^7^

### 2.6 Statistical analysis

Data are processed using GraphPad software and presented as the mean ± SD of results from at least three independent experiments. A Student t test was used for statistical comparison between means where applicable. Differences among more than two groups in the above assays were estimated using one‐way ANOVA. *P < .05; **P < .01.

## 3 RESULTS

### 3.1 NE (ELA2) induces the EMT within RLE‐6TN and A549 cells

**Figure 1:**
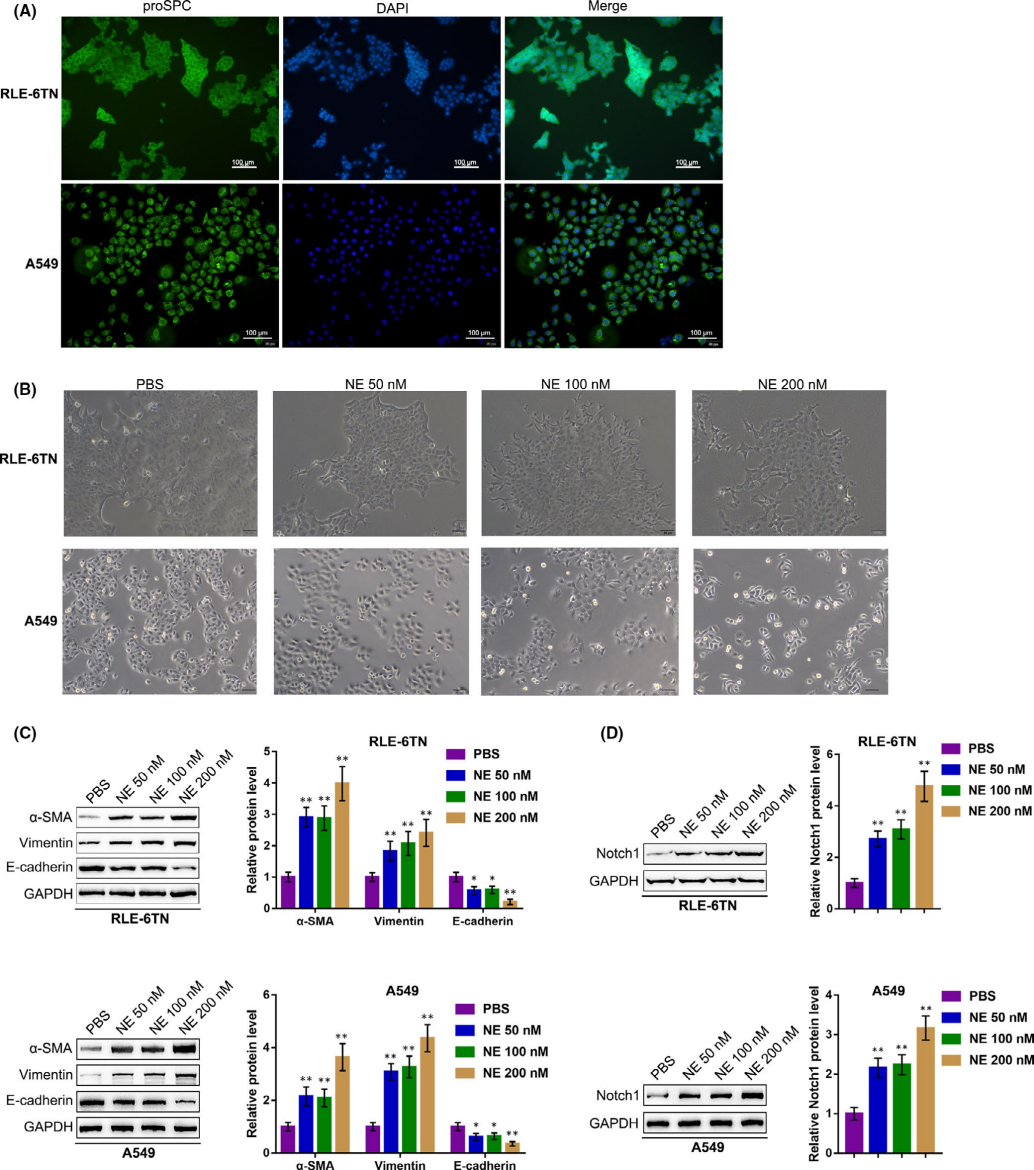
NE (ELA2) induces the epithelial‐mesenchymal transition (EMT) in RLE‐6TN and A549 cells. (A) RLE‐6TN and A549 cells were authenticated using immunofluorescence staining (IF staining) with anti‐proSPC. Next, the cells were treated with PBS, 50, 100 or 200 nM NE and examined for (B) the microscopic appearance; (C) the protein levels of α‐SMA, vimentin and E‐cadherin were determined using Immunoblotting; (D) the protein levels of Notch1 were determined using Immunoblotting. *P < .05, **P < .01

Before investigate the functions of NE in EMT and Notch1 cleavage in RLE‐6TN and A549 cells, the study first authenticated the lung epithelial cells by examining the alveolar epithelial type II cell marker pro‐surfactant protein C (proSPC) using IF staining; as shown in Figure 1A, strong expression of the alveolar epithelial type II cell marker proSPC was observed. Next, we treated the RLE‐6TN and A549 cells with PBS or 50, 100 or 200 nM NE and examined for the changes in the microscopic appearance and EMT markers. Under the microscope, the RLE‐6TN and A549 cells lost the round‐shape and NE treatment dose‐dependently induced the morphology of cells becoming fibroblast‐like spindle‐shaped (Figure 1B). Consistently, NE treatment dose‐dependently enhanced the protein contents of myofibroblast markers (mesenchymal markers), α‐SMA and vimentin, and suppressed the protein contents of epithelial marker, E‐cadherin (Figure 1C). As for the effects of NE treatment on Notch1 cleavage, NE treatment also dose‐dependently increased Notch1 protein levels (Figure 1D). These data suggest that NE treatment could induce EMT in RLE‐6TN and A549 cells; Notch1 signalling pathway could contribute to NE‐induced EMT.

### 3.2 NE induces Notch1 cleavage to enhance the EMT within RLE‐6TN and A549 cells

**Figure 2:**
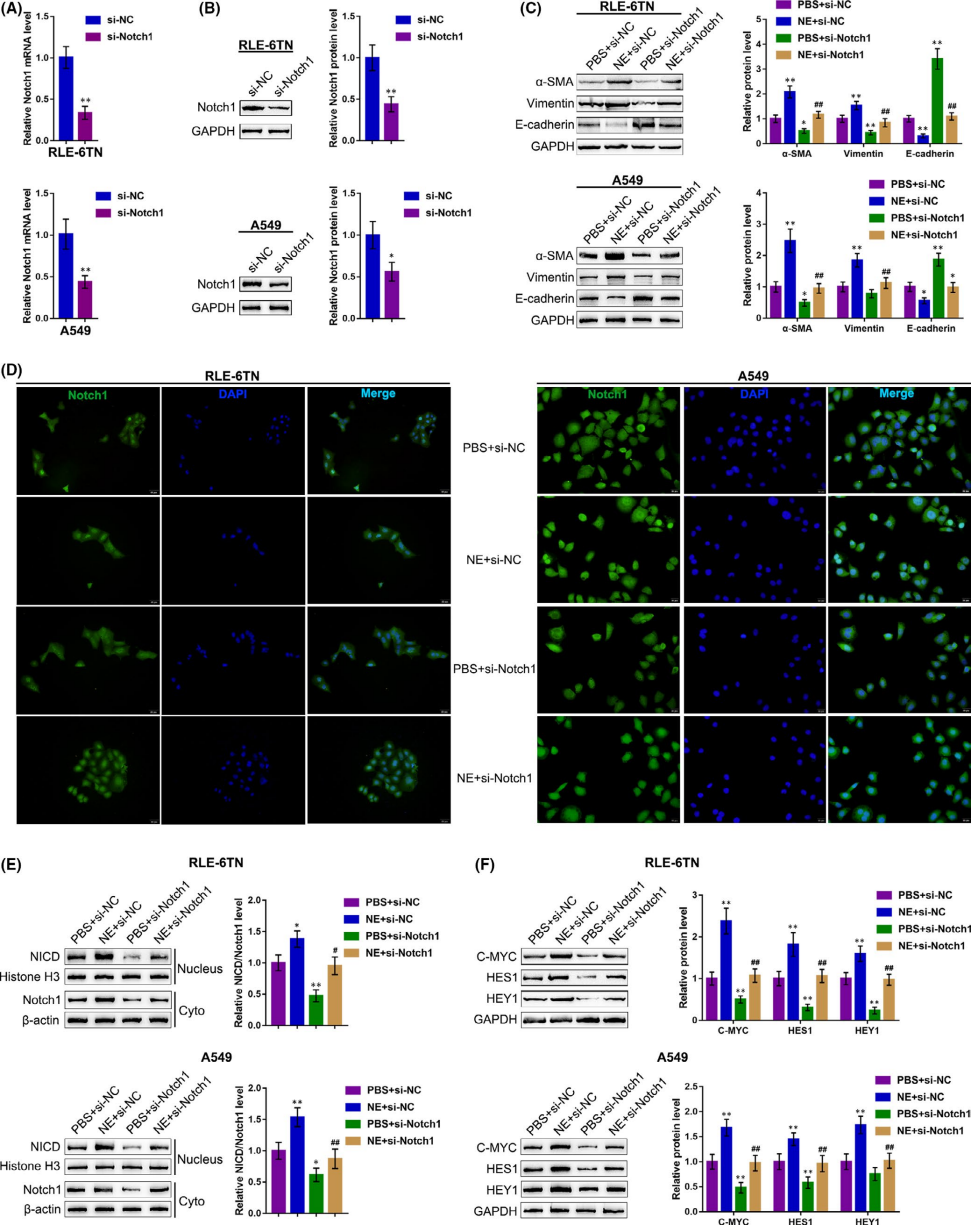
NE induces Notch1 cleavage to enhance the EMT in RLE‐6TN and A549 cells. Notch1 knockdown was generated in RLE‐6TN and A549 cells by transfection of si‐Notch1. The transfection efficiency was validated by (A) real‐time PCR and (B) Immunoblotting. Next, RLE‐6TN and A549 cells were transfected with si‐Notch1 under the treatment of PBS or NE and examined for (C) the protein levels of α‐SMA, vimentin and E‐cadherin were measured using Immunoblotting; (D) the protein content and the distribution of Notch1 were determined using IF staining; (E) the protein levels of NICD in nucleus and Notch1 in cytoplasm were determined using Immunoblotting; (F) the protein levels of Notch1, C‐MYC, HES1 and HEY1 were determined using Immunoblotting. *P < .05, **P < .01, compared to the control group; ##*P* < .01, compared to the PBS + si‐Notch1 group

To investigate the specific roles of Notch1 in NE‐induced EMT, we transfected si‐Notch1 to generate Notch1 knockdown within RLE‐6TN and A549 cells, then performed real‐time PCR (Figure 2A) and Immunoblotting (Figure 2B) to verify the transfection efficiency. Next, we transfected RLE‐6TN and A549 cells with si‐Notch1 under the treatment of PBS or NE and applied for further experiments. NE treatment significantly enhanced the protein levels of α‐SMA and vimentin and inhibited the protein level of E‐cadherin, while Notch1 knockdown exerted an opposing effect (Figure 2C); the effects of NE treatment on EMT markers were significantly reversed by Notch1 knockdown (Figure 2C). These data suggest that Notch1 indeed affects NE‐induced EMT within RLE‐6TN and A549 cells.

To investigate the underlying mechanism, we examined whether NE treatment could affect Notch1 cleavage and Notch1 intracellular cytoplasmic domain (NICD) nucleus translocation. As shown in Figure 2D, IF staining showed that NE treatment enhanced the fluorescence intensity representing Notch1 both in RLE‐6TN and A549 cell nucleus and cytoplasm; however, the enhancement showed to be more significant within the nucleus. On the contrary, Notch1 knockdown attenuated the fluorescence intensity representing Notch1. These data indicate that NE treatment induced NICD nuclear translocation. This was further evidenced by the Immunoblotting analysis that NE treatment increased, while Notch1 knockdown decreased the protein ratio of nucleus NICD/ cytoplasmic Notch1 (Figure 2E). Moreover, NE treatment also increased the protein levels of Notch1 and Notch1 cleavage downstream factors, including C‐MYC, HES1 and HEY1, while Notch1 knockdown decreased these protein levels. Notably, the above‐described effects of NE treatment were all significantly reversed by Notch1 knockdown (Figure 2F), indicating that NE enhances Notch1 cleavage and NICD nuclear translocation, therefore promoting EMT.

### 3.3 Effects of Emodin on NE‐induced Notch1 cleavage and EMT in RLE‐6TN and A549 cells

**Figure 3:**
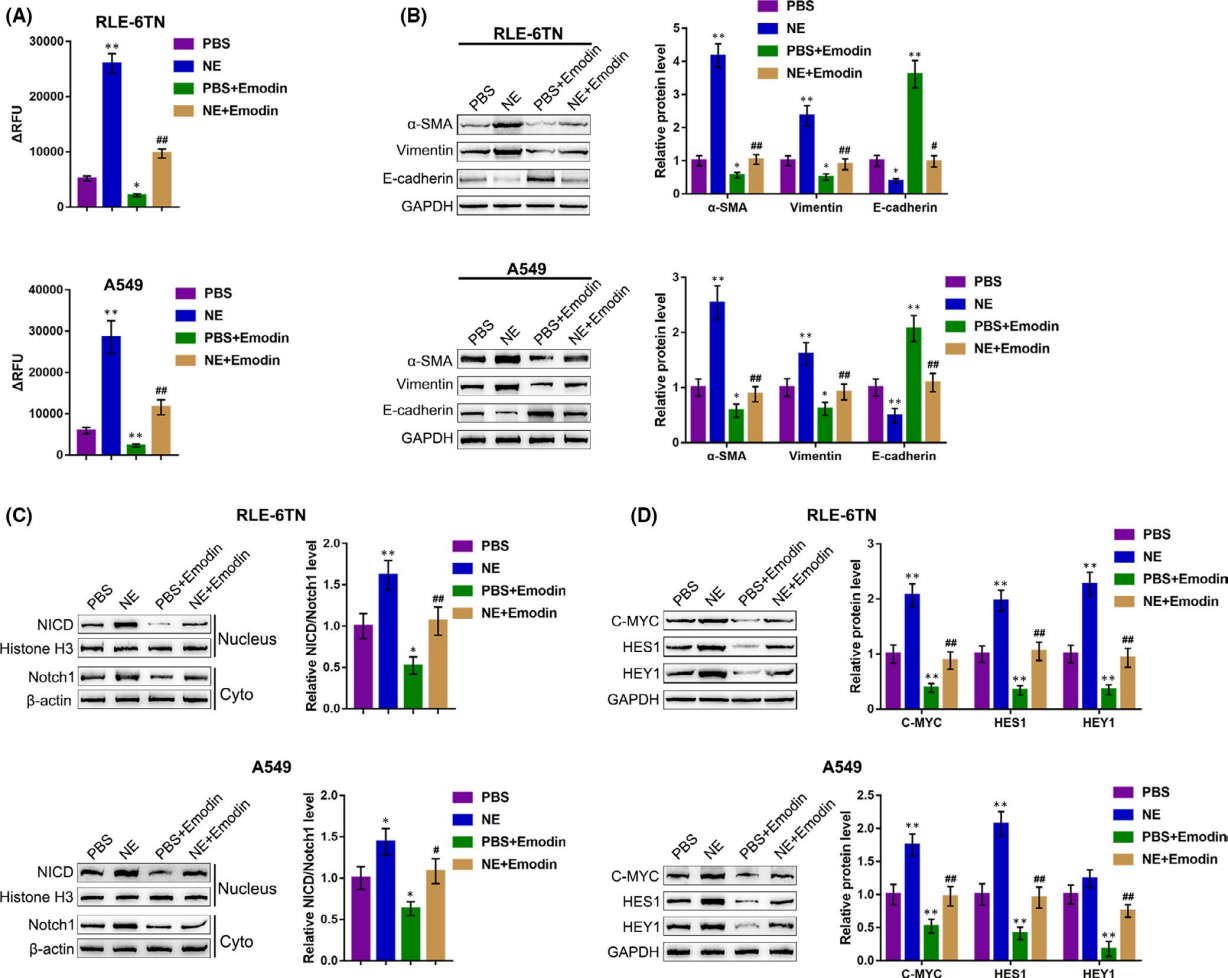
Effects of Emodin on NE‐induced Notch1 cleavage and EMT in RLE‐6TN and A549 cells. RLE‐6TN and A549 cells were treated with NE or co‐treated with NE and Emodin and examined for (A) the NE enzymatic activity using a Neutrophil Elastase Activity Assay Kit; (B) the protein levels of α‐SMA, vimentin and E‐cadherin were determined using Immunoblotting; (C) the protein levels of NICD in nucleus and Notch1 in cytoplasm were determined using Immunoblotting; (D) the protein levels of Notch1, C‐MYC, HES1 and HEY1 were determined using Immunoblotting. *P < .05, **P < .01, compared to the control group; #*P* < .05, ##*P* < .01, compared to the PBS + Emodin group

As previously reported, Emodin could inhibit the inducible effects of TGF‐β1 on Notch‐1 nuclear translocation within RLE‐6TN cells.^16^ Moreover, by screening previous experimental results from the online Protein‐Protein Interaction database, ChEMBL (www.ebi.ac.uk/chembl/) and BindingDB (www.bindingdb.org), we found that Emodin targets several proteins, including NE (ELA2) (Figure S1). Thus, the study speculates that Emodin affects NE‐induced Notch1 cleavage to modulate EMT within RLE‐6TN and A549 cells. To verify this speculation, we treated RLE‐6TN and A549 cells with NE or co‐treated with NE and Emodin and performed further analyses. Firstly, Emodin treatment significantly inhibited NE enzyme activity, which was partially reversed by NE treatment (Figure 3A). In the meantime, Emodin decreased the protein levels of α‐SMA and vimentin (mesenchymal markers), and increased the protein level of E‐cadherin (epithelial marker); the effects of Emodin on EMT markers were significantly reversed by NE treatment (Figure 3B). Regarding the Notch1 cleavage, Emodin inhibited NICD/Notch1 protein ratio, while NE treatment reversed the effects of Emodin on NICD/Notch1 protein ratio (Figure 3C). Consistently, Emodin also decreased the protein levels of Notch1 downstream factors, including C‐MYC, HES1 and HEY1, while NE treatment significantly reversed the effects of Emodin on these proteins (Figure 3D), indicating that Emodin inhibits NE‐induced Notch1 cleavage, therefore inhibiting EMT within RLE‐6TN and A549 cells.

### 3.4 Emodin inhibits NE enzyme activity to inhibit NE‐caused EMT within RLE‐6TN cells

**Figure 4:**
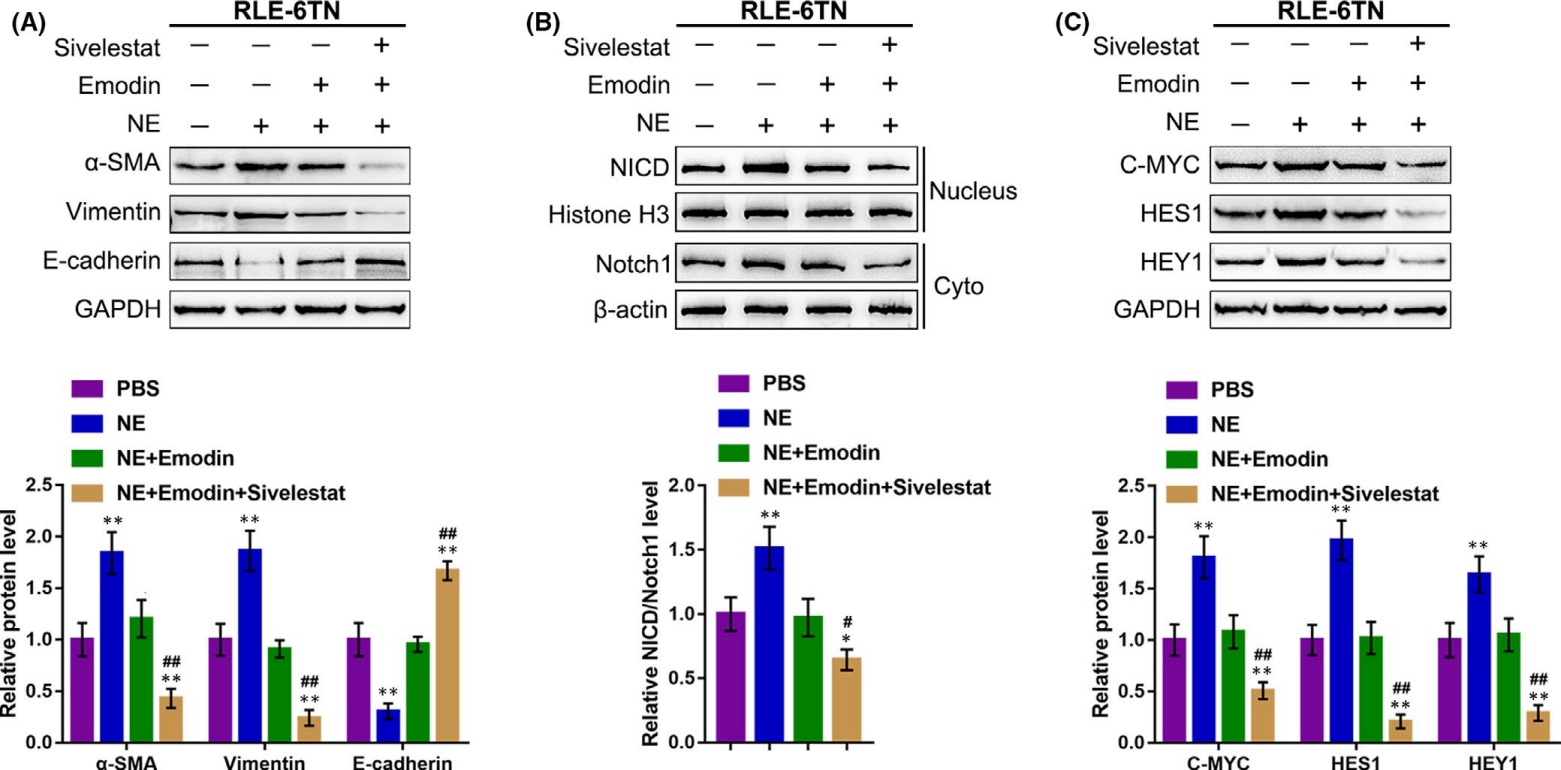
Emodin affects NE enzyme activity to modulate EMT in RLE‐6TN cells. RLE‐6TN cells were divided into four groups and treated with PBS, NE along, Emodin + NE, or Sivelestat + Emodin +NE and examined for (A) the protein levels of α‐SMA, vimentin and E‐cadherin using Immunoblotting; (B) the protein levels of NICD in nucleus and Notch1 in cytoplasm were measured using Immunoblotting; (C) the protein levels of C‐MYC, HES1 and HEY1 were measured using Immunoblotting. **P < .01, compared to the control group; ##*P* < .01, compared to the NE + Emodin group

To further confirm the above‐described findings, we divided RLE‐6TN cells into four groups and treated them with PBS, NE along, Emodin + NE or Sivelestat (a neutrophil elastase inhibitor) + Emodin + NE, respectively. As shown in Figure 4A, NE treatment up‐regulated the protein contents of α‐SMA and vimentin (mesenchymal markers) and decreased those of E‐cadherin (epithelial marker), whereas the effects of NE on EMT markers were remarkably attenuated by Emodin treatment and more attenuated by the combination of Emodin and Sivelestat (Figure 4A). Regarding the Notch1 cleavage, NE treatment remarkably enhanced the protein ratio of NICD/Notch1, and Notch1 cleavage downstream factors, including C‐MYC, HES1 and HEY1, while the inducible effects of NE on these proteins were attenuated by Emodin treatment and more attenuated by the combination of Emodin and Sivelestat (Figure 4B‐C). These data indicate that Emodin impairs the enzyme activity of NE, therefore inhibiting NE‐induced Notch1 cleavage and EMT in RLE‐6TN cells.

### 3.5 Inhibition of Notch1 pathway further enhances the effects of Emodin on NE‐induced EMT

**Figure 5:**
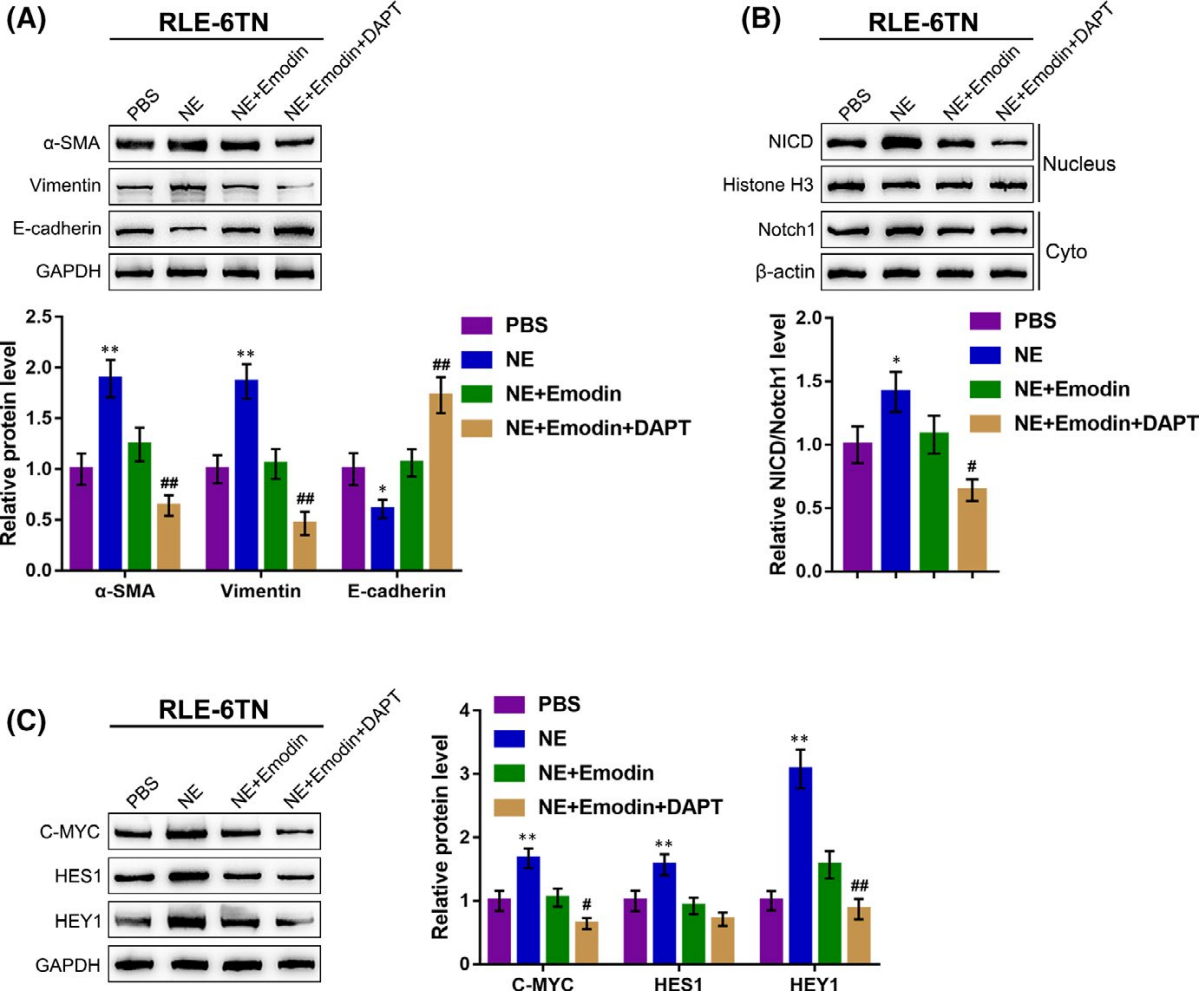
DAPT further enhanced the Effects of Emodin on NE‐induced EMT RLE‐6TN cells were divided into four groups and treated with PBS, NE along, Emodin + NE or DAPT + Emodin +NE and examined for (A) the protein levels of α‐SMA, vimentin and E‐cadherin using Immunoblotting; (B) the protein levels of NICD in nucleus and Notch1 in cytoplasm were measured using Immunoblotting; (C) the protein levels of C‐MYC, HES1 and HEY1 were measured using Immunoblotting. **P < .01, compared to the control group; ##*P* < .01, compared to the NE + Emodin group

To further investigate the role of Notch1 cleavage during the NE‐induced EMT process, we divided RLE‐6TN cells into four groups and treated them with PBS, NE along, Emodin + NE or DAPT (Notch1 inhibitor) + Emodin + NE, respectively. As shown in Figure 5A, NE treatment up‐regulated the protein contents of α‐SMA and vimentin, and decreased those of E‐cadherin, whereas the effects of NE on EMT markers were remarkably attenuated by Emodin treatment and more attenuated by the combination of Emodin and DAPT (Figure 5A). Regarding the Notch1 cleavage, NE treatment remarkably enhanced the protein ratio of NICD/Notch1, and Notch1 cleavage downstream factors, including C‐MYC, HES1 and HEY1, while the inducible effects of NE on these proteins were reduced by Emodin treatment and more reduced by the combination of Emodin and DPAT (Figure 5B‐C). These data indicate that Emodin inhibited NE‐induced EMT in RLE‐6TN cells via suppressing the Notch1 pathway activity (Notch1 cleavage).

## 4 DISCUSSION

Herein, NE treatment was observed to promote EMT, Notch1 cleavage and NICD nuclear translocation within RLE‐6TN and A549 cell lines. The promotive roles of NE treatment in these events were significantly reversed by Notch1 knockdown. Emodin treatment remarkably inhibited the enzyme activity of NE, suppressed EMT, Notch1 cleavage and NICD nuclear translocation within RLE‐6TN and A549 cells, while NE treatment significantly reversed the effects of Emodin. Moreover, the effects of NE on EMT, Notch1 cleavage and NICD nuclear translocation were remarkably attenuated by Emodin treatment and more attenuated by the combination of Emodin and Sivelestat or DAPT.

Neutrophils are involved in the pathogenic processing of both IPF (idiopathic pulmonary fibrosis) and bleomycin (BLM)‐induced pulmonary fibrosis.^26^, ^27^, ^28^ As an elastolytic enzyme with a wide range of substrates, neutrophil elastase (NE) can digest most proteins and appear to play a role in certain human pulmonary injury.^29^ It is known that NE level within BAL (bronchoalveolar lavage) fluides is increased in IPF, suggesting that NE elevation may be involved in the pathogenesis.^23^ In the present study, we first found that NE might be a target protein of Emodin, which might suppress the TGF‐β1‐caused EMT through inhibiting Notch1 nuclear translocation within alveolar epithelial cells, according to ChEMBL (www.ebi.ac.uk/chembl/) and BindingDB (www.bindingdb.org) Protein‐Protein Interaction databases. Regarding EMT, another group has reported that polymorphonuclear neutrophils or NE could induce the polygonal epithelial morphology of the ovarian cancer cells becoming spindle‐shaped, of which the most likely cause was membrane E‐cadherin degradation that led to loss of cellular contacts and polarity.^30^ Herein, we also observed the polygonal epithelial morphology of RLE‐6TN cells becoming spindle‐shaped. Moreover, the mesenchymal markers were increased while epithelial maker was decreased by NE treatment, indicating that NE treatment also induces EMT in alveolar epithelial cell lines.

In addition to EMT markers, NE treatment also dramatically up‐regulated the protein levels of Notch1, one membrane receptors of the Notch signalling. Notch elicits myofibroblast differentiation of alveolar epithelial cells via a TGF‐β‐Smad3 signalling pathway activating SMA gene transcription.^3^ It has been reported that Notch1 activation directly stimulates the expression of α‐SMA to induce myofibroblast differentiation.^31^ Notably, as previously reported, Emodin could regulate Notch pathway.^32^, ^33^ In the OVA‐induced asthma mice model, Notch1, Notch2 and Notch3 were all highly expressed in the lung tissues. In response to the OVA and Emodin treatment, Notch1 shows greater changes compared to Notch 2 and Notch 3 in the lung tissues.^34^ Similarly, in the present study, we also observed that Emodin down‐regulated Notch 2 and Notch 3 in the presence or absence of NE. However, the change in Notch1 protein level shows greater extent (Figure S2), suggesting that Notch1 may play the main role among other notch receptors. Moreover, we also found that Emodin down‐regulated Notch‐1 and C‐MYC expression to suppress EMT. In addition, as a Notch‐1‐specific inhibitor, DAPT obviously enhanced the function of Emodin upon Notch pathway and EMT.^16^ Herein, NE treatment significantly up‐regulated the protein contents of NICD, Notch1 cleavage downstream factors, including HES1, HEY1 and MYC, and the nuclear fluorescence intensity representing NICD. On the contrary, Notch1 knockdown within RLE‐6TN cells remarkably reversed the effects of NE treatment, indicating that NE might promote EMT within RLE‐6TN cells by promoting Notch1 cleavage and NICD nuclear translocation. Notably, Duan et al^35^ reported that a new, widely expressed gene, N2N, whose product is homologous to Notch 2, could interact with neutrophil elastase and is a target of NE. Moreover, they also figured out that co‐expressed N2N represses the transcriptional activities of both Notch1 and Notch2 intracellular domains, indicating that there is a functional relationship between N2N and Notch family proteins.^35^ Thus, it is reasonable to speculate that the effects of NE on the Notch1 signalling might also be related to the novel Notch protein N2N, which needs further investigation.

As revealed by ChEMBL (www.ebi.ac.uk/chembl/) and BindingDB (www.bindingdb.org) Protein‐Protein Interaction databases retrieval, NE is a target protein of Emodin, which might suppress TGF‐β1–induced EMT through inhibiting Notch1 nuclear translocation within RLE‐6TN cells in our previous study.^16^ Herein, we found that Emodin remarkably inhibited NE enzyme activity. More importantly, NE‐induced EMT and Notch1 cleavage were significantly attenuated by Emodin, and even more attenuated by the combination of Emodin and NE inhibitor Sivelestat. These data indicate that Emodin could inhibit NE enzyme activity, therefore suppressing NE‐induced Notch1 cleavage and EMT.

In conclusion, we revealed the involvement of NE‐induced Notch1 cleavage in the functions of Emodin suppressing NE‐caused EMT in RLE‐6TN cells. This novel mechanism of Emodin inhibiting EMT might extend the application of Emodin in PF treatment.

## CONFLICT OF INTEREST

The authors declare that there are no conflicts of interest.

## AUTHOR CONTRIBUTION

**Linshui Zhou:** Conceptualization (equal); Investigation (equal); Writing‐original draft (equal). **Rundi Gao:** Investigation (equal). **Huihua Hong:** Investigation (equal); Software (equal). **Xiaojuan Li:** Investigation (equal). **Jia Yang:** Data curation (equal); Writing‐review & editing (supporting). **Wei Shen:** Data curation (equal); Software (equal). **Zhen Wang:** Data curation (equal); Resources (equal). **Junchao Yang:** Conceptualization (equal); Project administration (equal); Supervision (equal).

## AUTHOR CONTRIBUTIONS

LinshuiZhou, Rundi Gao and Junchao Yang: Experimental design and supervising the whole experimental process; Huihua Hong and Xiaojuan Li: Experimental conducting; Jia Yang, Wei Shen and Zhen Wang: Data analysis and manuscript preparation. All the authors read, revised and approved the final manuscript.

## ETHICS APPROVAL AND CONSENT TO PARTICIPATE

Not Applicable.

## Supporting information

Fig S1

Fig S2

## Data Availability

Please contact the authors for data requests.

## References

[1] **Giri** SN . Novel pharmacological approaches to manage interstitial lung fibrosis in the twenty‐first century. Annu Rev Pharmacol Toxicol. 2003;43:73‐95. 12540741 10.1146/annurev.pharmtox.43.100901.135740

[2] **Zhang** K , **Rekhter** MD , **Gordon** D , **Phan** SH . Myofibroblasts and their role in lung collagen gene expression during pulmonary fibrosis. A combined immunohistochemical and in situ hybridization study. Am J Pathol. 1994;145:114‐125. 7518191 PMC1887314

[3] **Aoyagi‐Ikeda** K , **Maeno** T , **Matsui** H , et al. Notch induces myofibroblast differentiation of alveolar epithelial cells via transforming growth factor‐{beta}‐Smad3 pathway. Am J Respir Cell Mol Biol. 2011;45:136‐144. 21749980 10.1165/rcmb.2010-0140oc

[4] **Willis** BC , **Liebler** JM , **Luby‐Phelps** K , et al. Induction of epithelial‐mesenchymal transition in alveolar epithelial cells by transforming growth factor‐beta1: potential role in idiopathic pulmonary fibrosis. Am J Pathol. 2005;166:1321‐1332. 15855634 10.1016/s0002-9440(10)62351-6 PMC1606388

[5] **Dasgupta** SB , **Mukherjee** S . Old Wine in a New Bottle: harnessing the therapeutic properties of Emodin derivatives. Journal of Biomedical Engineering and Medical Devices. 2016; 2015.

[6] **Kuo** P‐C , **Hsieh** T‐F , **Lin** M‐C , **Huang** B‐S , **Huang** J‐W , **Huang** H‐C . Analysis of antifungal components in the galls of Melaphis chinensis and their effects on control of anthracnose disease of chinese cabbage caused by Colletotrichum higginsianum. J Chemist. 2015;2015.

[7] **Stein** RL . Catalysis by human leukocyte elastase: III. Steady‐state kinetics for the hydrolysis of p‐nitrophenyl esters. Arch Biochem Biophys. 1985;236:677‐680. 3844313 10.1016/0003-9861(85)90673-3

[8] **Artavanis‐Tsakonas** S , **Rand** MD , **Lake** RJ . Notch signaling: cell fate control and signal integration in development. Science. 1999;284:770‐776. 10221902 10.1126/science.284.5415.770

[9] **Gridley** T . Notch signaling in vascular development and physiology. Development. 2007;134:2709‐2718. 17611219 10.1242/dev.004184

[10] **Iso** T , **Kedes** L , **Hamamori** Y . HES and HERP families: multiple effectors of the Notch signaling pathway. J Cell Physiol. 2003;194:237‐255. 12548545 10.1002/jcp.10208

[11] **Xu** K , **Moghal** N , **Egan** SE . Notch signaling in lung development and disease. Adv Exp Med Biol. 2012;727:89‐98. 22399341 10.1007/978-1-4614-0899-4_7

[12] **Liu** T , **Hu** B , **Choi** YY , et al. Notch1 signaling in FIZZ1 induction of myofibroblast differentiation. Am J Pathol. 2009;174:1745‐1755. 19349363 10.2353/ajpath.2009.080618 PMC2671263

[13] **Timmerman** LA , **Grego‐Bessa** J , **Raya** A , et al. Notch promotes epithelial‐mesenchymal transition during cardiac development and oncogenic transformation. Genes Dev. 2004;18:99‐115. 14701881 10.1101/gad.276304 PMC314285

[14] **Leong** KG , **Karsan** A . Recent insights into the role of Notch signaling in tumorigenesis. Blood. 2006;107:2223‐2233. 16291593 10.1182/blood-2005-08-3329

[15] **Hu** B , **Wu** Z , **Bai** D , **Liu** T , **Ullenbruch** MR , **Phan** SH . Mesenchymal deficiency of Notch1 attenuates bleomycin‐induced pulmonary fibrosis. Am J Pathol. 2015;185:3066‐3075. 26358219 10.1016/j.ajpath.2015.07.014 PMC4630167

[16] **Gao** R , **Chen** R , **Cao** Y , et al. Emodin suppresses TGF‐beta1‐induced epithelial‐mesenchymal transition in alveolar epithelial cells through Notch signaling pathway. Toxicol Appl Pharmacol. 2017;318:1‐7. 27989784 10.1016/j.taap.2016.12.009

[17] **Albert** R , **DasGupta** B , **Dondi** R , et al. A novel method for signal transduction network inference from indirect experimental evidence. J Comput Biol. 2007;14:927‐949. 17803371 10.1089/cmb.2007.0015

[18] **Han** JD , **Bertin** N , **Hao** T , et al. Evidence for dynamically organized modularity in the yeast protein‐protein interaction network. Nature. 2004;430:88‐93. 15190252 10.1038/nature02555

[19] **Watts** DJ , **Strogatz** SH . Collective dynamics of 'small‐world' networks. Nature. 1998;393:440‐442. 9623998 10.1038/30918

[20] **Makino** T , **Gojobori** T . Evolution of protein‐protein interaction network. Genome Dyn. 2007;3:13‐29. 18753782 10.1159/000107601

[21] **Takemasa** A , **Ishii** Y , **Fukuda** T . A neutrophil elastase inhibitor prevents bleomycin‐induced pulmonary fibrosis in mice. Eur Respir J. 2012;40:1475‐1482. 22441751 10.1183/09031936.00127011

[22] **Taooka** Y , **Maeda** A , **Hiyama** K , **Ishioka** S , **Yamakido** M . Effects of neutrophil elastase inhibitor on bleomycin‐induced pulmonary fibrosis in mice. Am J Respir Crit Care Med. 1997;156:260‐265. 9230758 10.1164/ajrccm.156.1.9612077

[23] **Yamanouchi** H , **Fujita** J , **Hojo** S , et al. Neutrophil elastase: alpha‐1‐proteinase inhibitor complex in serum and bronchoalveolar lavage fluid in patients with pulmonary fibrosis. Eur Respir J. 1998;11:120‐125. 9543280 10.1183/09031936.98.11010120

[24] **Chua** F , **Dunsmore** SE , **Clingen** PH , et al. Mice lacking neutrophil elastase are resistant to bleomycin‐induced pulmonary fibrosis. Am J Pathol. 2007;170:65‐74. 17200183 10.2353/ajpath.2007.060352 PMC1762691

[25] **Roghanian** A , **Sallenave** JM . Neutrophil elastase (NE) and NE inhibitors: canonical and noncanonical functions in lung chronic inflammatory diseases (cystic fibrosis and chronic obstructive pulmonary disease). J Aerosol Med Pulm Drug Deliv. 2008;21:125‐144. 18518838 10.1089/jamp.2007.0653

[26] **Crystal** RG , **Bitterman** PB , **Rennard** SI , **Hance** AJ , **Keogh** BA . Interstitial lung diseases of unknown cause. Disorders characterized by chronic inflammation of the lower respiratory tract (first of two parts). N Engl J Med. 1984;310:154‐166. 6361560 10.1056/NEJM198401193100304

[27] **Crystal** RG , **Bitterman** PB , **Rennard** SI , **Hance** AJ , **Keogh** BA . Interstitial lung diseases of unknown cause. Disorders characterized by chronic inflammation of the lower respiratory tract. N Engl J Med. 1984;310:235‐244. 6361563 10.1056/NEJM198401263100406

[28] **Nagai** A , **Aoshiba** K , **Ishihara** Y , et al. Administration of alpha 1‐proteinase inhibitor ameliorates bleomycin‐induced pulmonary fibrosis in hamsters. Am Rev Respir Dis. 1992;145:651‐656. 1372163 10.1164/ajrccm/145.3.651

[29] **Doring** G . The role of neutrophil elastase in chronic inflammation. Am J Respir Crit Care Med. 1994;150:S114‐S117. 7952645 10.1164/ajrccm/150.6_Pt_2.S114

[30] **Mayer** C , **Darb‐Esfahani** S , **Meyer** AS , et al. Neutrophil Granulocytes in Ovarian Cancer ‐ Induction of Epithelial‐To‐Mesenchymal‐Transition and Tumor Cell Migration. J Cancer. 2016;7:546‐554. 27053953 10.7150/jca.14169 PMC4820731

[31] **Noseda** M , **Fu** Y , **Niessen** K , et al. Smooth Muscle alpha‐actin is a direct target of Notch/CSL. Circ Res. 2006;98:1468‐1470. 16741155 10.1161/01.RES.0000229683.81357.26

[32] **Deng** G , **Ju** X , **Meng** Q , **Yu** ZJ , **Ma** LB . Emodin inhibits the proliferation of PC3 prostate cancer cells in vitro via the Notch signaling pathway. Mol Med Rep. 2015;12:4427‐4433. 26081222 10.3892/mmr.2015.3923

[33] **Kim** J , **Lee** JS , **Jung** J , **Lim** I , **Lee** JY , **Park** MJ . Emodin suppresses maintenance of stemness by augmenting proteosomal degradation of epidermal growth factor receptor/epidermal growth factor receptor variant III in glioma stem cells. Stem Cells Dev. 2015;24:284‐295. 25229646 10.1089/scd.2014.0210 PMC4303020

[34] **Hua** S , **Liu** F , **Wang** M ; JMsmimjoe, research c . Emodin alleviates the airway inflammation of cough variant asthma in mice by regulating the notch pathway. Med Sci Monit. 2019;25:5621–5629. 31354164 10.12659/MSM.915080 PMC6685324

[35] Duan Z , Li FQ , Wechsler J , et al. A novel notch protein, N2N, targeted by neutrophil elastase and implicated in hereditary neutropenia. Mol Cell Biol. 2004;24(1):58–70. 10.1128/mcb.24.1.58-70.2004 14673143 PMC303357

