# Supplementary figures and images for: Emodin inhibiting neutrophil elastase‐induced epithelial‐mesenchymal transition through Notch1 signalling in alveolar epithelial cells
Source: J Cell Mol Med. 2020 Sep 15;24(20):11998–2007. doi: 10.1111/jcmm.15827 (PMC7578861; doi:10.1111/jcmm.15827)

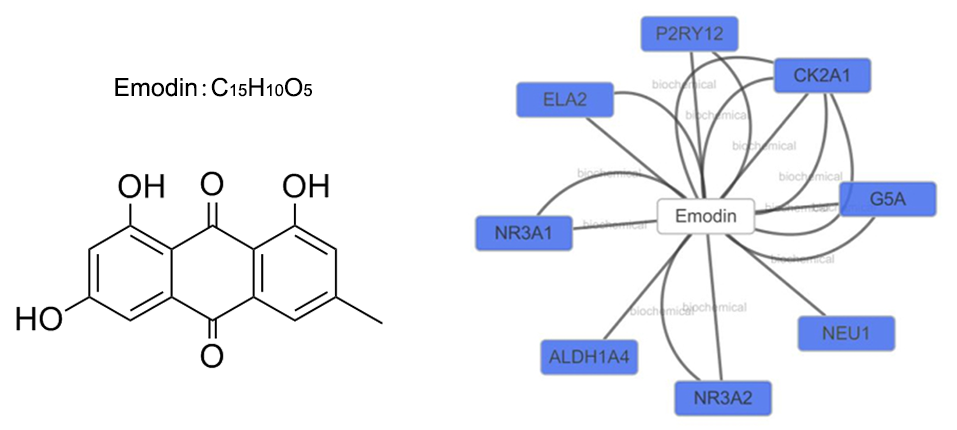

Supplement: Supplementary file 1 — Fig S1 [file JCMM-24-11998-s001.tif]

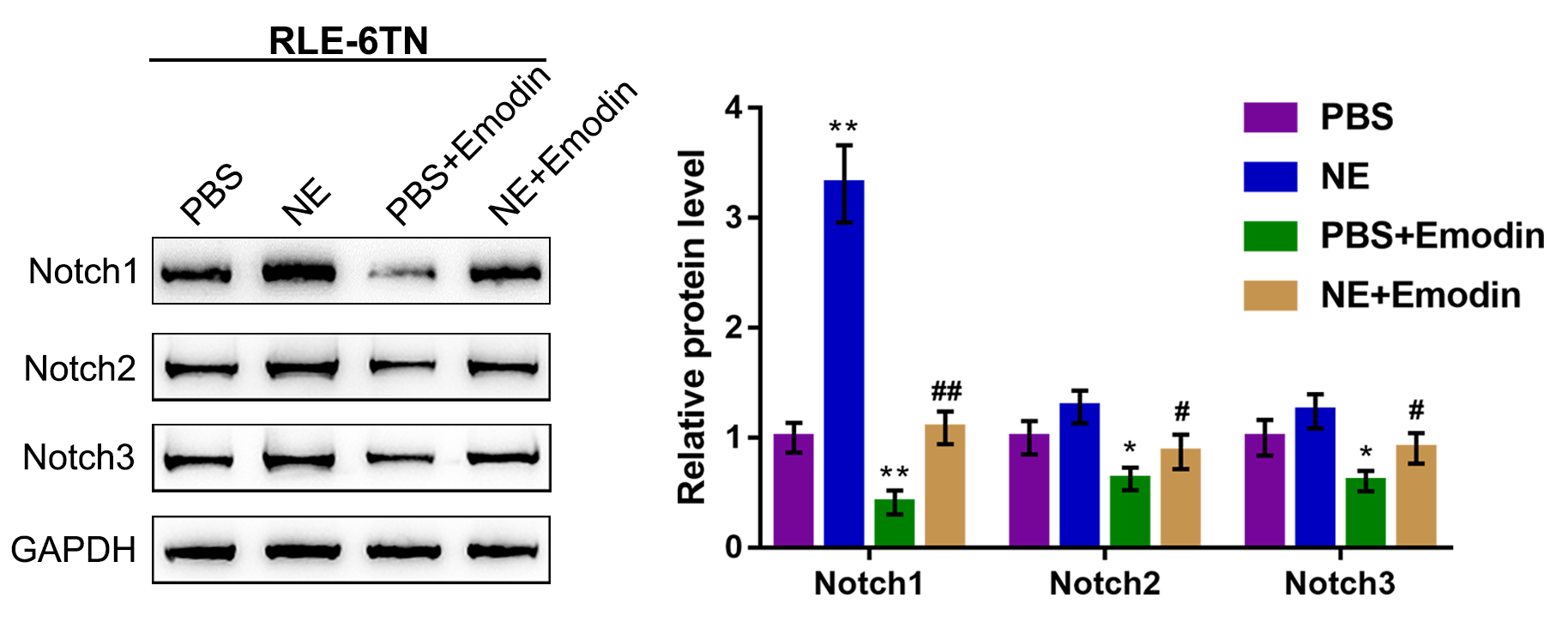

Supplement: Supplementary file 2 — Fig S2 [file JCMM-24-11998-s002.tif]
